# Electro-Magnetism, Etc.

**Published:** 1872-11

**Authors:** A. Donald

**Affiliations:** Louisiana


### ELECTRO-MAGNETISM IN CHRONIC DISEASES.

BY A. DONALD, M. D., OF LOUISIANA.

*Messrs. Editors:*—In answer to your request, as to the use of Electricity in the treatment of diseases, I have some experience and observation, having used it more or less for twenty-five years, and I am free to say that I have cured many cases of Chronic affections, that *I could not* have cured without it. I can see no reason why Electro-Magnetism should not be regarded as the vital force in the animal economy, since we can prove to a demonstration that it often performs the office of the vital force. In *all chronic* diseases, there is a want of vital force. This we assume to be true; and may it not be a fact that we often fail to cure the disease because we do not supply the necessary amount of the vital element? We wait upon the system to appropriate our tonics and stimulants, under the hope that they will supply and arouse the vital activity so as to induce a state of health, but how often is it that we see the case, under this course (no matter how well directed) going still lower? But since *nature moves slowly*, and continuously, can we not here learn a lesson, and imitate her laws, and where we find a want of vital force in a part, *there supply electricity often and slowly*.

In the vegetable kingdom we see this force acting *slowly;* first the germ, then the bud, the leaf, and the flower; and we observe *nothing hasty* in all this, but when we take a case of chronic disease, how often is it that even when electricity is used, it is done in so *much haste*, with only a *few* applications, and the patient is not cured, that the remedy is laid aside, as of no use? This is not according to natural law, and therefore, it is of no use. The following case will demonstrate what perseverance and patience may accomplish. On the 1st of January, 1869, I was called to see Mrs. S——, who was suffering with an enlarged spleen, of *nine years'* standing. It was *the largest* one that I had ever seen. She informed me that during that time several physicians had given her medicine for months, but that no benefit had resulted from the medicines taken. I took the case more for the experiment than with the belief that I should succeed. With one of Jerome Kidder's batteries, I commenced the application of electricity, by placing the positive pole on the nape of the neck, and made the connection by moving my right hand over the spleen, and continued one hour every day. I kept the bowels open with *one pill* given every other night, composed as follows: Podophyllin, gr. ½; Leptandrin, gr. i; Asclepin, gr. ½. Thus, in two months and a half, by daily applications of one hour each, and the use of the pill, I had the pleasure of seeing this lady cured of an enlarged spleen, which had resisted what was regarded as the best treatment in such cases.

I have reported this case to show that in some extremely low and morbid cases, that *something more* than the ordinary remedies of the Materia Medica, *is needed* to arouse vital action and restore the normal condition in a part.

If you should think my suggestions about *going slow and continuously* with electricity, and the report of the above case, worth anything, use it, otherwise cast it aside.

